# TreeExp2: An Integrated Framework for Phylogenetic Transcriptome Analysis

**DOI:** 10.1093/gbe/evz222

**Published:** 2019-10-14

**Authors:** Jingwen Yang, Hang Ruan, Wenjie Xu, Xun Gu

**Affiliations:** 1 MOE Key Laboratory of Contemporary Anthropology, School of Life Sciences, Fudan University, Shanghai, China; 2 Human Phenome Institute, Fudan University, Shanghai, China; 3 Department of Biochemistry and Molecular Biology, The University of Texas Health Science Center at Houston-McGovern Medical School; 4 Department of Genetics, Development and Cell Biology, Iowa State University

**Keywords:** transcriptome evolution, R-package, high-throughput analysis, phylogenomics

## Abstract

Recent innovations of next-generation sequencing such as RNA-seq have generated an enormous amount of comparative transcriptome data, which have shed lights on our understanding of the complexity of transcriptional regulatory systems. Despite numerous RNA-seq analyses, statistical methods and computational tools designed for phylogenetic transcriptome analysis and evolution have not been well developed. In response to this need, we developed software *TreeExp2* specifically for RNA-seq data. The R-package *TreeExp2* has implemented a suite of advanced, recently developed methods for transcriptome evolutionary analysis. Its main functions include the ancestral transcriptome inference, estimation of the strength of expression conservation, new expression distance, and the relative expression rate test. *TreeExp2* provides an integrated, statistically sound framework for phylogenetic transcriptome analysis. It will considerably enhance our analytical capability for exploring the evolution and selection at the transcriptome level. The current version of *TreeExp2* is available under GPLv3 license at the Github developer site https://github.com/jingwyang/TreeExp; last accessed November 12, 2019, and its online tutorial which describes the biological theories in details and fully worked case studies with real data can be found at https://jingwyang.github.io/TreeExp-Tutorial; last accessed November 12, 2019.

## Introduction

Recent remarkable progress in next-generation sequencing (RNA-seq) (Wang et al. 2009) has shed some lights on one of central topics in evolutionary biology, that is, how gene regulation plays a key role in phenotypic innovations (King and Wilson 1975; Lehner 2013). Although an enormous amount of transcriptome data from multiple tissues with diverse species have been generated (Brawand et al. 2011; Barbosa-Morais et al. 2012; McCarthy et al. 2012; Necsulea and Kaessmann 2014; Xu et al. 2018; Cardoso-Moreira et al. 2019), the challenge immediately becomes the availability of statistically sound analytical tools that enable evolutionists to explore the pattern of transcriptome evolution. In 1980s and 1990s, promoted by advances in DNA sequencing techniques, the conceptual framework of DNA sequence evolution and analytical methods had been well developed (Nei 1987, 2014; Nei and Kumar 2000; Yang 2006, 2014). For instance, a rich body of theoretical and empirical studies were published about the evolutionary distance between two sequences. However, the expression distance for transcriptome evolution between species has not been generally accepted among a number of distance measures proposed in the literature (Gu and Su 2007; Pereira et al. 2009; Gu et al. 2013; Sudmant et al. 2015; Chen and He 2016).

**Fig. 1.**
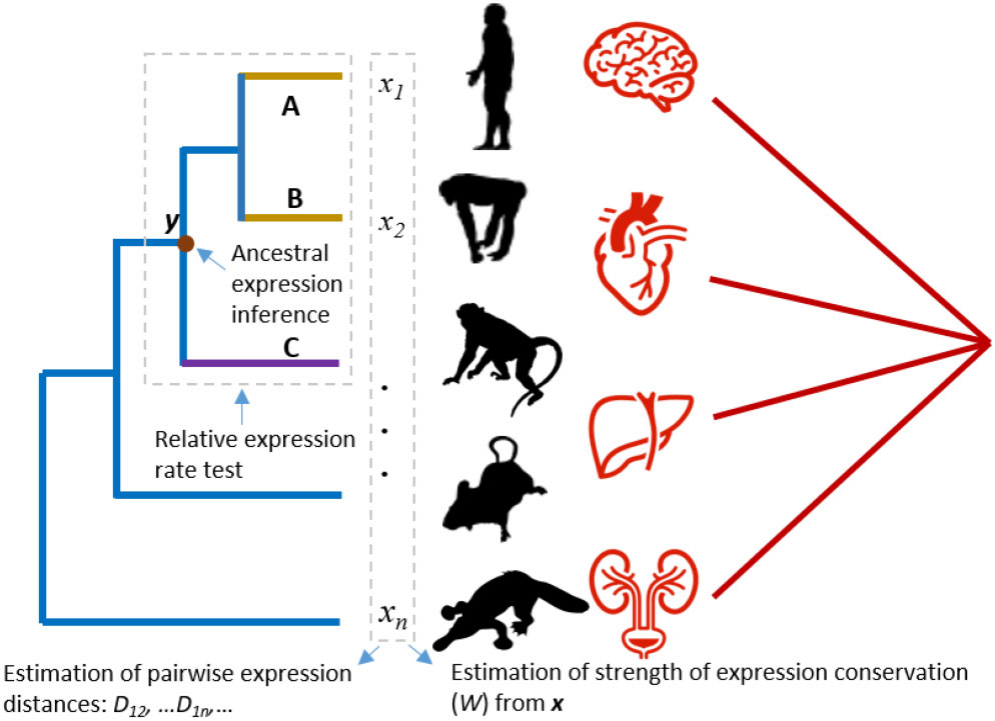
—RNA-seq data from multiple species and tissues, illustrated by expression levels ***x**** *=* *(*x*_1_, *x*_2_, …, *x_n_*) of an orthologous gene over *n* species. *TreeExp2* can perform the following analyses. 1) Infer the ancestral expression state (node *y* in red brown as example) of a gene in a tissue, which is a (phylogeny-dependent linear) combination of ***x***. 2) Estimate the strength of expression conservation (*W*) for any gene when ***x*** is given in a tissue. 3) Calculate expression distance that is linear to the evolutionary time. And 4) detect lineage-specific fast-evolving expression divergence in species *A* or *B* (yellow branches) using species *C* (purple branch) as outgroup.

Apparently, development of an integrated, statistically sound framework for the evolutionary transcriptome analysis is highly desirable. It is interesting to have a comparison with MEGA (Kumar et al. 2008, 2018), a widely used software package for molecular evolutionary genetics analysis. Yet, we fully acknowledge that phylogenetic analysis for transcriptome evolution is still in the early stage comparing to phylogenetic analysis of DNA and protein sequence. Moreover, the theoretical foundation of transcriptome to describe how gene expression evolves between species has not been well developed. As a first attempt, in this article, we present an R-package, *TreeExp2*, which is designed to make several published (Gu 2016; Gu et al. 2017, 2019; Yang et al. 2018) and new statistical methods available for phylogenetic analysis of transcriptome data. Based on our previous work in developing bioinformatics tools implemented in *TreeExp* 1.0 (*Tree*-dependent *Exp*ression analysis for short) (Ruan et al. 2016), the *TreeExp2* is the updated version with a suite of advanced, recently developed methods. The package can be applied to comparative expression evolution analysis based on RNA-seq data, which includes pairwise expression distance estimation, relative rate test for transcriptome evolution, the strength of expression conservation estimation, ancestral transcriptome inference, etc. Figure 1 illustrates the main features and functions in *TreeExp2*, as well as supplementary table S1, Supplementary Material online, for a summary. The current version of *TreeExp2* is available under GPLv3 license at the Github developer site https://github.com/jingwyang/TreeExp; last accessed November 12, 2019, and its online tutorial which describes the biological theories in detail and fully worked case studies with real data can be found at https://jingwyang.github.io/TreeExp-Tutorial; last accessed November 12, 2019.

## Results and Discussion

Overall, *TreeExp2* offers an analytical framework under a unified evolutionary model to help our understanding of transcriptome evolution that may underlie phenotypic evolution across species. Several new features are discussed below. One may also see the tutorial document that not only describes the statistical model in details but also demonstrates each method by the real data set. To become more flexible when this R-package is applied to a broad range of research projects with various experimental designs and data types, *TreeExp2* only adopted the normalized expression data set as input, without any specific requirement for the normalization procedure. Nevertheless, we strongly recommend users should consider their multispecies input data sets to be processed appropriately so that they are comparable between tissue and species samples. RPKM (Reads Per Kilobase Million) or FPKM (Fragments Per Kilobase Million) method has been widely used to normalize the raw reads count data and to remove the feature-length and library size effects; the drawback is that this procedure tends to be less stable when the number of expressed genes differs considerably across samples. This problem can be mostly (if not all) alleviated by the TPM (Transcripts Per Kilobase Million) method that can effectively normalize the differences in composition of the transcripts. More statistically sophisticated methods such as TMM (Trimmed Mean of *M*-values) and the median ratio normalization are also suggested (Robinson et al. 2010).

### The Stationary Ornstein–Uhlenbeck Model of Transcriptome Evolution

**Fig. 2.**
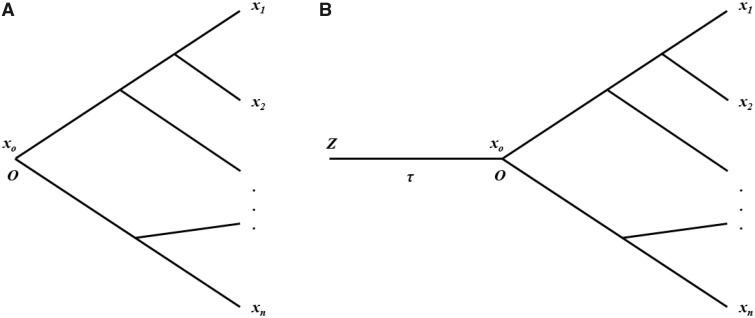
—The evolutionary phylogeny for comparative transcriptome analysis (*A*) under Ornstein–Uhlenbeck (OU) model. (*B*) Phylogeny when considering the origin of the tissue (node *Z*) to the root (node *O*) of the species tree. When the tissue origin is so ancient that *τ *→* *∞, it is called the stationary OU model along the species phylogeny.

Those new methods we have implemented in *TreeExp2* are based on the Ornstein–Uhlenbeck (OU) model that considers the stabilizing selection as the baseline model of transcriptome evolution (Hansen and Martins 1996; Lemos et al. 2005). The notion of optimal expression claims that stabilizing selection, which maintains the optima under the background of random mutations, dominates the transcriptome evolution (Bedford and Hartl 2009). Following the most common practice, the stabilizing selection on the expression of a gene (*x*) satisfies a Gaussian-like fitness,

(1) $$f(x)=e^{w{\left(x-\mu \right)}^{2}/2},$$

where *μ* is the optimal value and *w* is the coefficient of stabilizing selection; a large *w* means a strong selection pressure, and *vice versa*. Lande (1976) showed that the evolution of *X* follows an OU stochastic process (fig. 2*A*). That is, given the initial expression value *x*_0_, the OU model predicts that *x*(*t*), the values of *X* after *t* evolutionary time units, follows a normal distribution with the following mean *E*[*x*|*x*_0_] and variance *V*(*x*|*x*_0_):

(2) $$\begin{array}{c} E[x|x_{0}]\;=\;x_{0}e^{-\beta t}+\mu (1-e^{-\beta t})\\ V(x|x_{0})\;=\;\frac{1-e^{-2\beta t}}{W}, \end{array}$$

respectively, where the rate of expression evolution *β = Wσ*^2^, and *W = *2*N*_e_*w*; *N*_e_ is the effective population size (Hansen and Martins 1996; Bedford and Hartl 2009). Hence, an OU model can be concisely represented by OU(*x*|*x*_0_; *t*) (model parameters omitted for simplicity). Intuitively speaking, an OU process can be thought of as adding an elastic spring to a Brownian motion. As random mutations push the gene expression farther away from this fixed optimum, the strength of elastic return increases proportionally.

The stationary OU model along a given phylogeny can be described as follows. Consider the evolution from the origin of the tissue (node *Z*) to the root (node *O*) of the species tree (fig. 2*B*): The first part is the conventional species tree with a specified root (*O*), and the second part is the evolutionary lineage from the origin of the tissue (node *Z*) to the root (node O) of the species tree, with *τ* time units. Although the timing of tissue origin was so ancient that the root of species phylogeny (node *O*) can be approximated by the stationary condition as *τ *→* *∞, the mean and the variance of *x*_0_ at the root of phylogeny is simply given by *μ* and 1/*W*, respectively, according to equation (2). Since then, both the optimal level (*μ*) and the strength of stabilizing selection (*W*) remain constant along the species phylogeny. Consequently, the expression variances in all internal and external nodes are the same, which equal to 1/*W*. It has been shown that the stationary assumption can simplify the analysis considerably, because the variance–covariance matrix ***V*** along a phylogeny is root independent (Hansen and Martins 1996).

### New Method for Expression Distance

For phylogenetic transcriptome analysis, it is desirable to estimate the expression distance that is linear in evolutionary time (*t*), a property that most measures may not have (Sudmant et al. 2015). For two species diverged *t* time units ago, let *x*_1_ and *x*_2_ be the expression levels of an orthologous gene pair, respectively. Under the stationary OU model, it has been shown that the covariance between *x*_1_ and *x*_2_ is given by

(3) $$\text{Cov}\left(x_{1},\;x_{2}\right)=e^{-2\beta t}/W$$

and the variances Var(*x*_1_)* *=* *Var(*x*_2_)* *=* *1/*W.*Equation (3) indicates that the expression covariance between two species decays exponentially with time *t*, characterized by the expression distance defined by *D*_12_* *=* *2*βt*, where *β* is the rate of transcriptome evolution. Based on equation (3), it appears that this linear-to-time expression distance between species can be simply estimated by

(4) $$D_{12}=-\text{ln}\left(1-P_{12}\right),$$

where *P*_12_* *=* *1* *−* r*_12_ is the Pearson distance (*r*_12_ is the Pearson coefficient of correlation). Because the expression distance may vary considerably among different gene sets, it is important to evaluate the bias caused the gene selection procedure such as “only expressed genes included” or “all genes included.”

It should be noticed that equation (4) assumes that the optimal expression level (*μ*) is the same among genes, referred as the constant-*μ* distance. Because the optimal expression level (*μ*) actually varies considerably among genes, this assumption is biologically unrealistic. Indeed, computer simulations showed that neglecting the *μ*-variation among genes could lead to an underestimation of *D*_12_ by equation (4), which becomes nontrivial when *D*_12_* *>* *0.5. To correct the bias caused by the constant-*μ* assumption, we developed a new method called the variable-*μ* method, in which a general formula to estimate the express distance *D*_12_* *=* *2*βt* is given by

(5) $$D_{12}=-\text{ln}\left[\left(r_{12}-\pi \right)/\left(1-\pi \right)\right]=-\text{ln}\left[1-P_{12}/\left(1-\pi \right)\right]$$

(see Materials and Methods), where *π* measures the degree of *μ*-variation among genes: *π* = 0 means a constant-*μ* assumption and equation (5) reduced to equation (4), whereas *π* = 1 means a very strong *μ* variation among genes. *TreeExp2* implemented a statistical method to estimate the parameter *π*.

We estimated *π* = 0.35–0.40 based on mammalian RNA-seq data of six tissues (Brawand et al. 2011). In particular, table 1 presents a detailed analysis of transcriptome evolution between the human and macaque. The coefficient of expression correlation between species ranges from 0.71 to 0.90 among six tissues. We then estimated the expression distance *D*_12_ by three methods, that is, the Pearson distance, the constant-*μ* distance and the variable-*μ* distance. For instance, *D*_12_ of tissue liver is 0.104, 0.110, and 0.191, respectively, illustrating that different estimation methods may result in as many as 2-fold differences. If one assumes that the human–macaque split time about 29 Ma, the rate of expression evolution in liver is around 1.79 × 10^−9^–3.29 × 10^−9^ per year.

**Table 1:** Expression Distance Estimates between Human and Macaque (*t *=* *29 Ma)

Tissues	Brain	Cerebellum	Liver	Kidney	Heart	Testis
*r*_12_	0.901* *±* *0.004	0.893* *±* *0.004	0.896* *±* *0.004	0.876* *±* *0.005	0.708* *±* *0.007	0.744* *±* *0.004
Pearson expression distance						
Expression distance	0.089* *±* *0.004	0.107* *±* *0.004	0.104* *±* *0.004	0.124* *±* *0.005	0.292* *±* *0.007	0.256* *±* *0.004
Rate of transcriptome evolution	1.53* *×* *10^−9^	1.84* *×* *10^−9^	1.79* *×* *10^−9^	2.14* *×* *10^−9^	5.03* *×* *10^−9^	4.41* *×* *10^−9^
Constant-*μ* expression distance, equation (4)						
Expression distance	0.104* *±* *0.005	0.113* *±* *0.006	0.110* *±* *0.006	0.132* *±* *0.007	0.345* *±* *0.021	0.296* *±* *0.011
Rate of transcriptome evolution	1.79* *×* *10^−9^	1.95* *×* *10^−9^	1.90* *×* *10^−9^	2.28* *×* *10^−9^	5.95* *×* *10^−9^	5.10* *×* *10^−9^
Variable-μ expression distance, equation (5)						
Estimated *π*	0.354	0.384	0.401	0.377	0.327	0.392
Expression distance	0.148* *±* *0.008	0.191* *±* *0.009	0.191* *±* *0.009	0.222* *±* *0.010	0.569* *±* *0.038	0.547* *±* *0.023
Rate of transcriptome evolution	2.41* *×* *10^−9^	3.29* *×* *10^−9^	3.29* *×* *10^−9^	3.83* *×* *10^−9^	9.81* *×* *10^−9^	9.43* *×* *10^−9^

### Ancestral Transcriptome Inference along a Phylogeny

To trace the route of transcriptome evolution, ancestral transcriptome inference plays an essential role. We (Yang et al. 2018) recently reported a new statistically sound method particularly designed for high-throughput RNA-seq data. This phylogeny-dependent method used an empirical Bayesian approach under the OU model, which includes the Brownian motion model (Gu 2004) as a special case. Although the procedure is technically sophisticated, the biological interpretation is actually straightforward. Let ***x**** *=* *(*x*_1_, …, *x_n_*) be the observed expression profile of a given orthologous gene over *n* species, and *y* be the expression level at an ancestral node of interest. Yang et al. (2018) showed that the posterior mean of *y* conditional of *x*_1_, …, *x_n_* is given by

(6) $$y|x=E[y|x_{1},\ldots ,x_{n}]=b_{0}+\sum_{i=1}^{n}b_{i}x_{i},$$

where *b*_0_ and *b_i_* (*i *=* *1, …, *n*) are those coefficients specifically related to the ancestral node *y*, which are phylogeny dependent. Hence, Bayesian ancestral expression inference by equation (6) can be also interpreted as a simple linear combination of the current expression profile, weighted by node-specific coefficients. *TreeExp2* implemented a practically feasible algorithm to calculate the coefficients *b*_0_, *b*_1_, …, *b_n_*, which makes a fast reconstruction of ancestral transcriptomes.

### Estimation of the Strength (*W*) of Expression Conservation

Evolution of gene expression across species is subject to the stabilizing selection to maintain the optimal expression level. Although it is wildly accepted that the resulting expression conservation varies considerably among genes, statistically reliable estimation remains a challenge, due to very few species and a high number of unknown parameters. We (Gu et al. 2019) developed a gamma distribution model to describe the variation of the strength of expression conservation (*W*) among genes. Given the high-throughput RNA-seq data sets from multiple species, we then formulate an empirical Bayesian procedure to estimate *W* for any gene-*k* with the expression profile (***x***_*k*_) among *n* species, which can be concisely written by

(7) $$W|x_{k}=a/\left[c+Q\left(x_{k}\right)\right],$$

where *Q*(***x***_*k*_) is the quadratic function of gene-*k* after accounting for the phylogeny dependence of *n* species under study; two constants *a* and *c* can be estimated from the data. Because *Q*(***x***) measures the level of expression variability among species, equation (7) shows that a low expression variability among species indicates a strong strength of expression conservation (a large *W*-estimate), and *vice versa*. Actually, this property can be intuitively demonstrated in the case of star tree such that *Q*(***x***_*k*_)* *=* *Σ_*j*_(*x_k,_*_*j*_* − μ_k_*)^2^, where *μ_k_* is the mean expression level of gene-*k* among *j *=* *1, …, *n* orthologous genes. Our case studies showed that those *W*-estimates are useful to study the pattern of expression conservation during the species evolution. One frequently asked question is whether unexpressed genes should be included in this analysis. Our suggestion is as follows: 1) the all-gene set would give an objective picture of the *W*-estimates, regardless of no expression, low expression or high expression and 2) the expressed-gene set would allow researchers to focus on some interesting patterns that may relate to the phenotypic evolution, but it might be subjective to define unexpressed genes in multiple species. Our recommendation is to carry out the analysis for both treatments, and then compare between them. We believe it would become more informative.

### Phylogeny-Dependent Expression Distance Analysis

We implemented several tools for phylogeny-dependent expression distance analysis. 1) When an expression distance matrix from a set of species is calculated, we are able to infer the expression tree by the Neighbor-Joining method. The statistical reliability of the inferred expression tree can be further evaluated by the implemented bootstrapping approach by resampling orthologous genes with replacements. Comparative analysis between the expression tree and the sequence tree may help resolve the question to what extent the phylogenetic signals is maintained in the across-species transcriptome data. In *TreeExp2*, one may intuitively use the bootstrapping method to evaluate whether the different bifurcations in the tree topology is statistically meaningful. In the future, we shall develop a more powerful approach based on the minimum evolution principle to statistically discriminate between the star tree (no phylogenetic signal), the reference tree (correct phylogenetic signals) and expression tree (incorrect phylogenetic signals). 2) When the species phylogeny is biologically known or can be reliably inferred, it is useful to map the expression distances onto the given tree to further explore the pattern of expression divergence. A least squares algorithm is implemented, which estimates all branch lengths of the given tree topology by minimizing the summed squared deviations from the expression distances. 3) When studying the evolutionary pattern of multiple functionally related tissues, a phylogenetic network approach may be more suitable (Gu 2016; Gu et al. 2017). In the case of two-species/two-tissues quartet, one may have two internal branches that represent the expression divergences of developmental similarity (*γ*_D_) and evolutionary relatedness (*γ*_E_) which can be estimated.

A new application of phylogeny-dependent expression distance analysis is to test whether the rate of transcriptome evolution for a given gene set differs significantly between the lineages of species *A* and *B*, using a third species (*C*) as outgroup (fig. 1). Let *D_AB_*, *D_AC_*, and *D_BC_* be the pairwise expression distances, respectively. Because lineages *A* and *B* have experienced the same evolutionary time (*t*), the relative expression rate test considers the following statistic:

(8) $$\Delta_{\mathrm{AB}}=D_{\mathrm{AC}}-D_{\mathrm{BC}}.$$

The null hypothesis Δ_*AB*_* *=* *0 means an equal rate of expression divergence between lineages *A* and *B*. Rejection of this null indicates a lineage-specific rapid expression evolution. *TreeExp2* implemented a statistical method to determine the significance level. The relative expression rate test may have a broad applications for detecting the underlying mechanism of transcriptome evolution (Enard et al. 2002; Gilad et al. 2006).

In *TreeExp2*, implementation of the distance method through the popular Neighbor-Joining algorithm for expression phylogeny inference is straightforward, as long as the pairwise distance matrix is calculated. One may implement the parsimony method easily when the expression level of a gene has been classified into a few discrete states; the simplest one, for instance, is binary (expressed or not expressed). Because under the OU model the expression profile along a phylogeny follows a multivariate normal distribution, the maximum likelihood method can be implemented by searching the tree topology with the highest likelihood value, though the algorithm could be complicated. In the future, we will implement these phylogeny inference methods.

### Concluding Remarks

High-throughput, high dimension transcriptome data have considerably accelerated our studies of genome-wide expression profiles in a multitude of cell-types, tissues, and species. To our best knowledge, *TreeExp2* provides a unique toolkit to explore the pattern of transcriptome evolution ultimately toward the gene regulatory network level. Indeed, our preliminary analysis of transcriptome data in primate brain areas (Xu et al. 2018) has demonstrated the powerfulness of *TreeExp2* to detect fast-evolution coexpression modules in the human lineage (J. Yang and X. Gu, unpublished data).

## Materials and Methods

### Variable-*μ* Method for Expression Distance

Suppose that the optimal expression value *μ* varies among genes according to a normal distribution with the mean zero and variance *V_μ_*. Under the stationary OU model, one can show that the expression variances are given by *V*_11_* *=* V*_22_* *=* *1/*W *+* V_μ_ = V_T_* and the covariance by

(9) $${\text{Cov}}_{12}=e^{-2\beta t}/W+V_{\mu }.$$

By the definition of Pearson coefficient of correlation *r*_12_, we have

(10) $$r_{12}=(e^{-2\beta t}/W+V_{\mu })/(1/W+V_{\mu })=\;\pi +\left(1-\pi \right)e^{-2\beta t},$$

where *π *=* V_μ_*/*V_T_*. One can easily obtain equation (5) from equation (10).

When equation (5) is applied to the evolutionary analysis of RNA-seq data, we have to know the parameter *π*, which can be estimated when RNA-seq data of the same tissue from more than three (*n *≥* *3) species are available.

## Supplementary Material

Supplementary data are available at *Genome Biology and Evolution* online.

## Supplementary Material

evz222_Supplementary_Data
