# Supplementary material for: TreeExp2: An Integrated Framework for Phylogenetic Transcriptome Analysis
Source: Genome Biol Evol. 2019 Oct 14;11(11):3276–82. doi: 10.1093/gbe/evz222 (PMC6934891; doi:10.1093/gbe/evz222)
Supplement: evz222_Supplementary_Data [file evz222_supplementary_data.docx]

**TableS1.** A summary of main features implemented in *TreeExp2*

| **Features** | **Brief Description** |
| --- | --- |
| **Data storage and manipulation** | 1. Load in the expression data and wrap it in a *taxaExp* object. 2. Generate a sub-expression level table from a *taxaExp* object |
| **Expression distance estimation** | 1. Estimate the pairwise expression distance matrix from a *taxaExp* object by a specified distance method. 2. Estimate the pairwise expression distances under (stationary) sOU model when the expression optima is a constant among genes; see Eq.(4) 3. Estimate the pairwise expression distance under sOU model when optima varies among genes; see Eq.(5). 4. Infer the expression tree by the neighbor-joining method. 5. Bootstrapping method for evaluating the reliability of the inferred expression tree. |
| **Ancestral transcriptome inference** | 1. Calculate the inversed variance-covariance matrix from expression profiles across species. 2. Infer ancestral expression profile of each gene in each internal node of a phylogeny; see Eq.(6). |
| **Strength of expression conservation (*W*) estimation** | 1. Calculate an inversed correlation matrix between expression profiles of species. 2. Estimate parameters under the *Gamma* distribution model for the variation of the strength of expression conservation (*W*); see Eq.(7). 3. Calculate the quadratic quantity Q of each gene based on expression levels across species. 4. Calculate genes-specific *W* based on the empirical Bayesian procedure expression conservation. |
| **Relative rate test for transcriptome evolution** | 1. Statistical detection of lineage-specific rapid expression evolution of a given gene set; see Eq.(10). |
